# Foreign Items

**Published:** 1868-12-15

**Authors:** 


### *FOREIGN ITEMS.*

Ovariotomy.—M. Kœberle has reported to the Academy of Sciences, Paris, the statistical results of sixty-nine cases of ovariotomy, as follows:

1st. Considered with reference to adhesions.

The proportion of deaths of cases presenting no adhesions was one-seventh.

Of those presenting slight adhesions, one-fifth.

Of those with extensive adhesions one-half.

No operation having been left uncompleted.

These results agree very closely with those of Spencer Wells (of London), his proportions being one-sixth, one-fifth and one-half respectively. Six per cent. of his operations having been left uncompleted in consequence of extensive adhesions.

2nd. The gravity of the operation is proportional to the loss of blood.

3rd. The mortality has been exactly proportional to the duration of the operative proceedings.

4th. The causes and the ratio of mortality were as follows: septicæmia, 10.14; peritonitis, 10.14; peritonitis and septicæmia, 8.7; intestinal strangulation, enteritis, and intestinal tympanitis, each 1.45.

5th. The duration of life, in twenty-four fatal cases, was as follows: In one case, one day; in five cases, two days; in seven cases, three days; in four cases, four days; in one case, six days; in three cases, seven days; in two, eight days; and in one instance, one month after the operation.

6th. In thirteen cases both the ovaries were removed simultaneously, and in two of these the uterus was also extirpated, together with the ovaries; of these there were seven cures, and six deaths.

7th. The ages of the patients ranged from seventeen to sixty-two; the largest proportion of successful cases being comprised between the ages of thirty and thirty-five years. Above the age of fifty the mortality being five-sevenths.

8th. Adhesions to the abdominal parietes, to the epiploon, and to the intestine, were most frequently encountered in the successful cases. Those to the pelvis, especially those to the uterus, having occasioned a more considerable mortality, as has been also the result of adhesions to the liver and the mesentery.

9th. In reference to the operation of paraceutesis, the results show, that in those cases in which it had not been performed, the mortality was one-third, whilst after one such operation it was but one-fourth; and six cases, which had been twice tapped, all recovered.

Of those who had been subjected to this operation from three to eight times, the mortality was great. Of three cases, in which *Iodine* injections were used, one only recovered.

10th. The mortality was proportional to the length of the incision.

11th. The mortality was proportional to the weight of the tumors.

12th. Chloroformic vomiting had no influence in the cases without adhesions, whilst its influence upon the mortality of grave cases was very marked.

13th. The mortality has been notably diminished, by reason of improvements in operative proceedings lately introduced.

Cholera Morbus. — M. Chevalier has presented to the Academy of Medicine (Paris), a communication upon the production of this malady by the eating of ices in summer.

M. Shönbein (of Bâle, Switzerland) has discovered, and prepared, a test paper for the detection of *Hydrocyanic Acid*, even when present in the most extreme atomic division.

Goitre Cretinism. — M. Garrigou, consulting physician at the Mineral Springs of Aix, asserts that these endemics are due to the existence of *Magnesia*, and more especially the silicate of that earth, in the soil, which modifies all organisms, both vegetable and animal. His doctrine is based upon extensive observations made in the districts of the Pyrenees, where these maladies are endemic, and are coincident with such geological constitution.

M. Berlin (France) reports twenty-four new observations upon the therapeutic effects of irritating injections of *Chloride of Sodium*, *Nitrate of Silver*, and *Tincture of Iodine*, into the interior of morbid tissues. The solution of *Chloride of Sodium* was used at its maximum of concentration, that of *Nitrate of Silver* in the proportion of one-fifth, and the *Tincture of Iodine* was prepared according to the following formula: Distilled water, 40 grammes; *Iodide of Potassium*, 1 gramme; *Tincture of Iodine*, 10 grammes. The results obtained were the following, viz.:

I. Tumors formed by the development of the thyroid, 8; cures, 5; improvement, which might be considered as cures, 1; insignificant improvements and negative results, 2.

II. Tumors formed by the development of the lymphatic ganglia, of which there were two having a tendency to suppuration, 3; cures, 3.

III. Tumor formed by the development of a serous bursa, 1; cure, 1.

IV. Neuralgias, more especially sciatic, 7.

1st. Old sciatica treated with the silver injection, 2; cures, 2.

2nd. Old sciatica treated with salt water, 2; cures, 0.

3rd. Recent sciatica treated with salt water, 4; cures, 2.

4th. Recent sciatica treated with the silver injection, 1; cure, 1.

